# Effects of basic and dual-task training programs on physical function in Parkinson’s disease: The PARKEX study

**DOI:** 10.1371/journal.pone.0358534

**Published:** 2026-09-18

**Authors:** Juan Carlos Magaña, Silvia Enríquez-Calzada, Roger Prat, Cláudia M. Deus, Susana P. Pereira, Mercè Avellanet, Elvira Gea, Ariadna Laguna, Marta Martinez-Vicente, Ona Perez-Larumbe, Maria Giné-Garriga, Jorge Hernández-Vara, Joel Montane

**Affiliations:** 1 Universitat Ramon Llull, Facultat de Psicologia, Ciències de l’Educació i de l’Esport Blanquerna, Barcelona, Spain; 2 Grup de Malalties Neurodegeneratives de la Vall d’Hebron. Vall d’Hebron Institut de Recerca (VHIR), Barcelona, Spain; 3 MIA-Portugal, Multidisciplinary Institute of Ageing, University of Coimbra, Coimbra, Portugal; 4 CIBB, Center for Innovative Biomedicine and Biotechnology, University of Coimbra, Coimbra, Portugal; 5 UCIBIO, Applied Molecular Biosciences Unit, Department of Chemistry, Faculty of Science and Technology, NOVA University Lisbon, Caparica, Portugal; 6 Associate Laboratory i4HB-Institute for Health and Bioeconomy, Faculty of Science and Technology, NOVA University Lisbon, Caparica, Portugal; 7 Hospital Nostra Senyora de Meritxell, Escaldes-Engordany, Andorra; 8 Universitat d’Andorra, Sant Julià de Lòria, Andorra; 9 Aligning Science Across Parkinson’s (ASAP) Collaborative Research NetworkChevy Chase, Maryland, United States of America; 10 Institut de Neurociències-Autonomous Universitat de Barcelona (INc-UAB), Cerdanyola del Vallès, Spain; 11 Universitat Ramon Llull, Facultat de Ciències de la Salut Blanquerna, Barcelona, Spain; 12 Departament de Neurologia. Hospital Universitari Vall D’Hebron, Barcelona, Spain; Shandong University, CHINA

## Abstract

Exercise is a promising non-pharmacological intervention for motor and non-motor symptoms in Parkinson’s disease (PD). However, direct comparisons between basic physical training (BPT) and dual-task training remain limited. The objective of the current clinical trial is to evaluate clinical and functional effects of BPT and a BPT combined with dual-task functional exercises (BPT + FE) in individuals with PD. In this randomized controlled trial, 24 participants with idiopathic PD were allocated to BPT, BPT + FE, or a no-intervention control group (Con). Interventions lasted 12 weeks (three 60-minute sessions/week). Outcomes included quality of life (PDQ-39), depressive symptoms (BDI), lower-limb strength (1-minute Sit-to-Stand Test, STS), and functional mobility (Timed Up and Go, TUG). Group effects were analyzed using permutation-based ANOVA with Bonferroni correction. Both interventions significantly improved physical performance vs. controls. STS gains were observed for BPT vs. Con (Mean Difference, MD = 31.00; p = .0026) and BPT + FE vs. Con (MD = 19.00; p = .0026). TUG times improved for BPT vs. Con (MD = –2.07s; p = .0039) and BPT + FE vs. Con (MD = –2.43s; p = .0028). Although BDI and PDQ-39 did not show statistically significant changes between group differences (r_rb = –0.429 and –0.339, respectively), several subscales demonstrated medium effect sizes suggesting potentially meaningful trends: emotional well-being (BPT: r_rb = –0.428; BPT + FE: r_rb = –0.446), bodily discomfort (BPT: r_rb = –0.524), and activities of daily living (BPT + FE: r_rb = –0.429). In conclusion, both programs improved physical performance in PD. Patient-reported outcomes did not reach statistical significance, but effect-size patterns suggest possible psychosocial benefits that warrant confirmation in larger trials.

Trial registration

ClinicalTrials.gov, NCT05963425.

## Introduction

Parkinson’s disease (PD) is one of the most prevalent and disabling neurodegenerative disorders globally, and represents a substantial and growing burden on public health systems [1]. As of 2021, approximately 11.8 million individuals worldwide were living with PD, a number projected to exceed 12 million by 2040 and to reach 25.2 million by 2050, due to population aging and increased life expectancy [2]. The burden of PD is increasing faster than that of almost any other neurological disorder, placing increasing pressure on global health systems [3]. This rising burden underscores the urgent need for effective interventions targeting not only symptom relief but also disease-modifying mechanisms [4]. In addition to its well-known motor symptoms, PD also imposes a significant burden through non-motor symptoms such as depression, cognitive impairment, or compromised quality of life, which often remain under-recognized and undertreated.

Currently, there are no pharmacological treatments capable of modifying the course of the disease or controlling its neurodegenerative progressions, and available therapies are primarily symptomatic. In this context, physical activity (PA) has emerged as a promising non-pharmacological therapeutic approach capable of producing clinically meaningful improvements in PD and it is increasingly recognized as an effective strategy for delaying disease progression [5,6]. At the same time, there is growing interest in tailoring exercise interventions to individual patient profiles, optimizing their intensity, duration, and cognitive demands to maximize both adherence and therapeutic benefit. Recent research suggests that regular PA may not only alleviate motor symptoms but also confer potential neuroprotective effects by enhancing neuroplasticity, upregulating neurotrophic factors, and reducing neuroinflammation [7]. Moreover, PA can improve non-motor symptoms including mood disturbances, sleep disorders, and cognitive decline, thereby contributing to overall quality of life [8]. Given its multifaceted benefits and low risk profile, integrating structured exercise programs into standard care should be a crucial strategy for PD management; however, high-quality randomized controlled trials in PD populations remain limited, especially those comparing different types of exercise programs and their impact on standard clinical outcomes such as functional mobility, depressive symptoms, and quality of life. Previous studies have shown that dual-task functional training, which simultaneously challenges motor and cognitive functions, can provide greater improvements in gait, balance, and executive functioning compared to single-task approaches in people with PD [9].

Given the close interaction between motor and cognitive systems, targeting both domains together offers advantages beyond those achieved with motor training alone. Randomized controlled trials have implemented dual-task interventions combining locomotor activities with concurrent cognitive tasks (e.g., mental arithmetic, verbal fluency) or coordinated motor demands (e.g., obstacle negotiation, manipulative tasks), reporting improvements in mobility, balance, and selected executive domains such as divided attention, inhibitory control, working memory, and set-shifting [10–15].

The present study evaluated the clinical and functional outcomes of the PARKEX randomized controlled trial (NCT05963425) [16], which compared Basic Physical Training (BPT) and BPT combined with Functional Exercises (BPT + FE) against a no-intervention control group in patients with early-stage PD. The PARKEX trial was originally designed to evaluate changes in mitochondrial function as its primary biological endpoint. In addition to these mechanistic outcomes, clinical, motor, non-motor, and quality-of-life measures were prospectively collected to explore the potential functional impact of the intervention. We hypothesized that both interventions would lead to significant improvements in health-related quality of life, depressive symptoms, and functional mobility, with additional benefits expected from dual-task functional training components. This study aims to contribute to the growing body of evidence supporting structured PA as a key element in early PD management. It further explores the added value of dual-task functional training in enhancing physical and mental health outcomes, while providing practical guidance for the development of personalized and scalable exercise interventions for this population.

## Methods

### Study design

This randomized study employed a between-subjects experimental design to assess the effects of different interventions on quality of life, depressive symptoms, and functional performance. Participants were categorized into three groups based on the intervention received: (1) Basic Physical Training (BPT) focused on strength and resistance, (2) BPT combined with Functional Exercises (BPT + FE), corresponding to a dual-task training condition in which motor exercises were performed simultaneously with structured cognitive tasks, and (3) a no-intervention control group (Con). The 60-minute sessions were conducted 3 times a week for 3 months with 8 participants *per* group. Attendance was recorded at each session by the supervising trainer.

Outcome assessments were conducted by evaluators who were not involved in the intervention delivery.

Participant recruitment started in September 2023, and concluded in October 2023. The intervention phase and assessments for outcome measures concluded in May 2024. All study participants provided written informed consent prior to enrollment.

The trial was registered at ClinicalTrials.gov (NCT05963425) and the full clinical study protocol was previously published [16].

### Participants

A total of 24 individuals diagnosed with idiopathic PD were recruited by the Neurodegenerative Diseases Group of the Vall d’Hebron Research Institute (VHIR), Barcelona, Spain. The sample size (N = 24) was calculated based on the primary mitochondrial endpoint of the trial, as detailed in the published protocol. Clinical and functional outcomes analyzed in the present manuscript were secondary endpoints and the study was not specifically powered to detect changes in these measures. The inclusion criteria comprised patients diagnosed with idiopathic PD in early stages (H&Y stages I-III) during the ‘on’ phase, with good cognitive function (Montreal Cognitive Assessment (MoCA) score ≥26), aged between 45 and 75 years, on a stable medication regimen for at least four weeks prior to enrollment, and capable to participate in exercise programs. Exclusion criteria included comorbidities contraindicating exercise, cognitive impairment (with a MoCA score <26), and participation in other clinical trials.

Participants were randomly assigned by an independent investigator to the 3 groups (BPT, BPT + FE, or Con) using a computer-generated block randomization sequence (performed with the program Research Randomizer), and stratified by age and sex after checking the eligibility criteria. Sample size was calculated as described in the study protocol [16].

**Fig 1:**
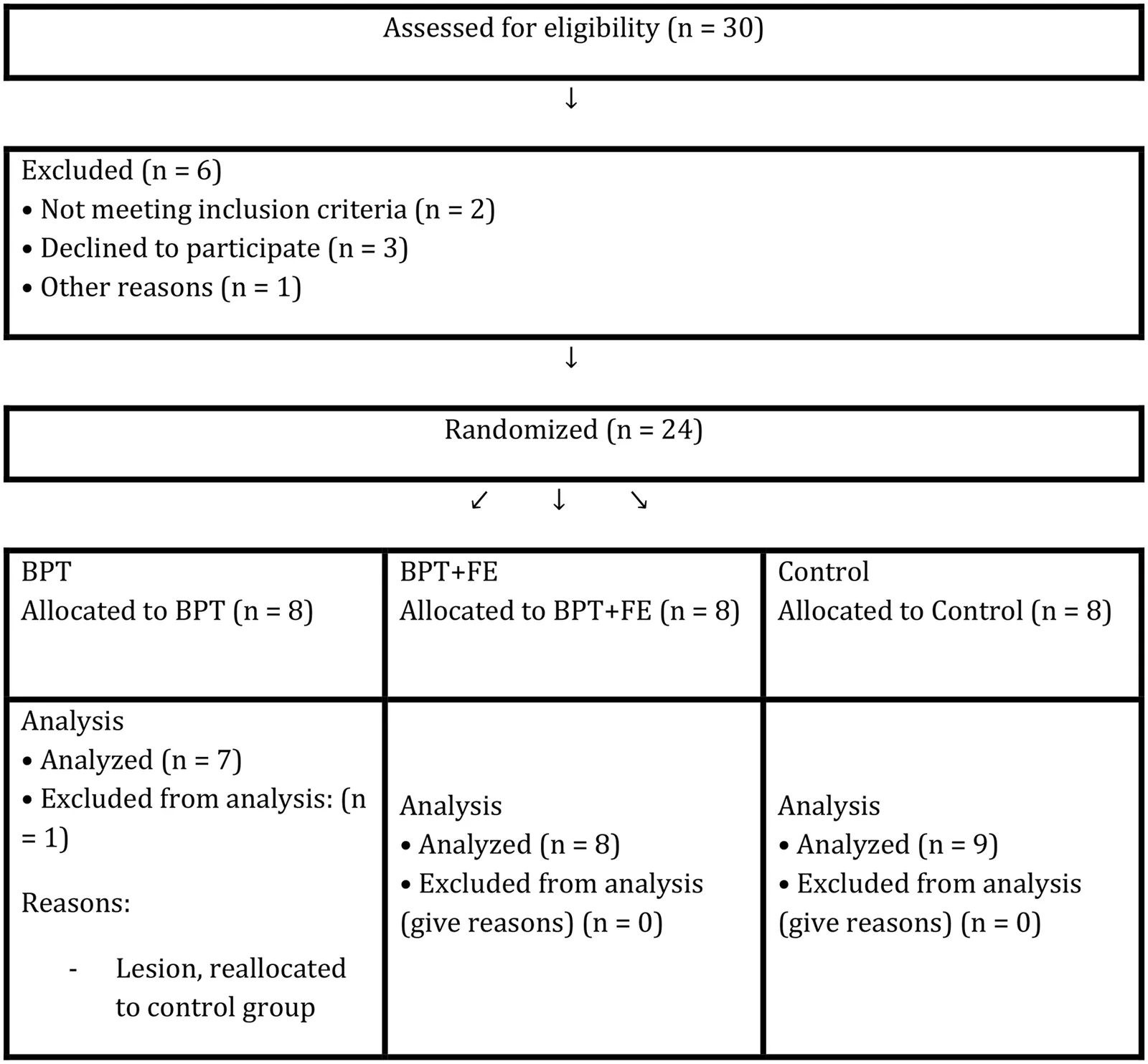
CONSORT flow diagram showing participant flow through the three study arms: BPT, BPT + FE, and control.

A CONSORT flow diagram illustrating participant recruitment, allocation, follow-up, and analysis is provided in Fig 1.

### Patient and public involvement

Patients and the public were not involved in the design or conduct of the study. However, patients contributed to the dissemination of the research findings by participating in conference presentations and related outreach activities. The study protocol was developed by clinicians and physical activity researchers.

### Overview of the Programs

In both intervention programs (BPT and BPT + FE), the core strength–endurance component (BPT) was delivered using eccentric resistance training with a flywheel device (*kBox4*, Exxentric AB, Stockholm, Sweden). The *kBox4* inertial discs available were: XS 0.005, S 0.010, M 0.025, and L 0.050 kg·m2, selected by movement pattern and postural demand.

The interventions were delivered 3 times per week for 12 weeks. While the overall session structure remained consistent, task complexity and training load were progressively increased based on participant performance.

The program was divided into four mesocycles of variable duration (ranging from 2 to 4 weeks), beginning with a neuromuscular adaptation phase, and progressing toward higher eccentric load and power-oriented stimuli. Training intensity was prescribed using the Borg CR10 scale, initially targeting a moderate level (Borg 3–5) and progressively increasing to high levels (Borg 7–9) [17]. Perceived effort was monitored every two weeks to ensure alignment with the intended intensity range and to guide individualized progression.

**Fig 2:**
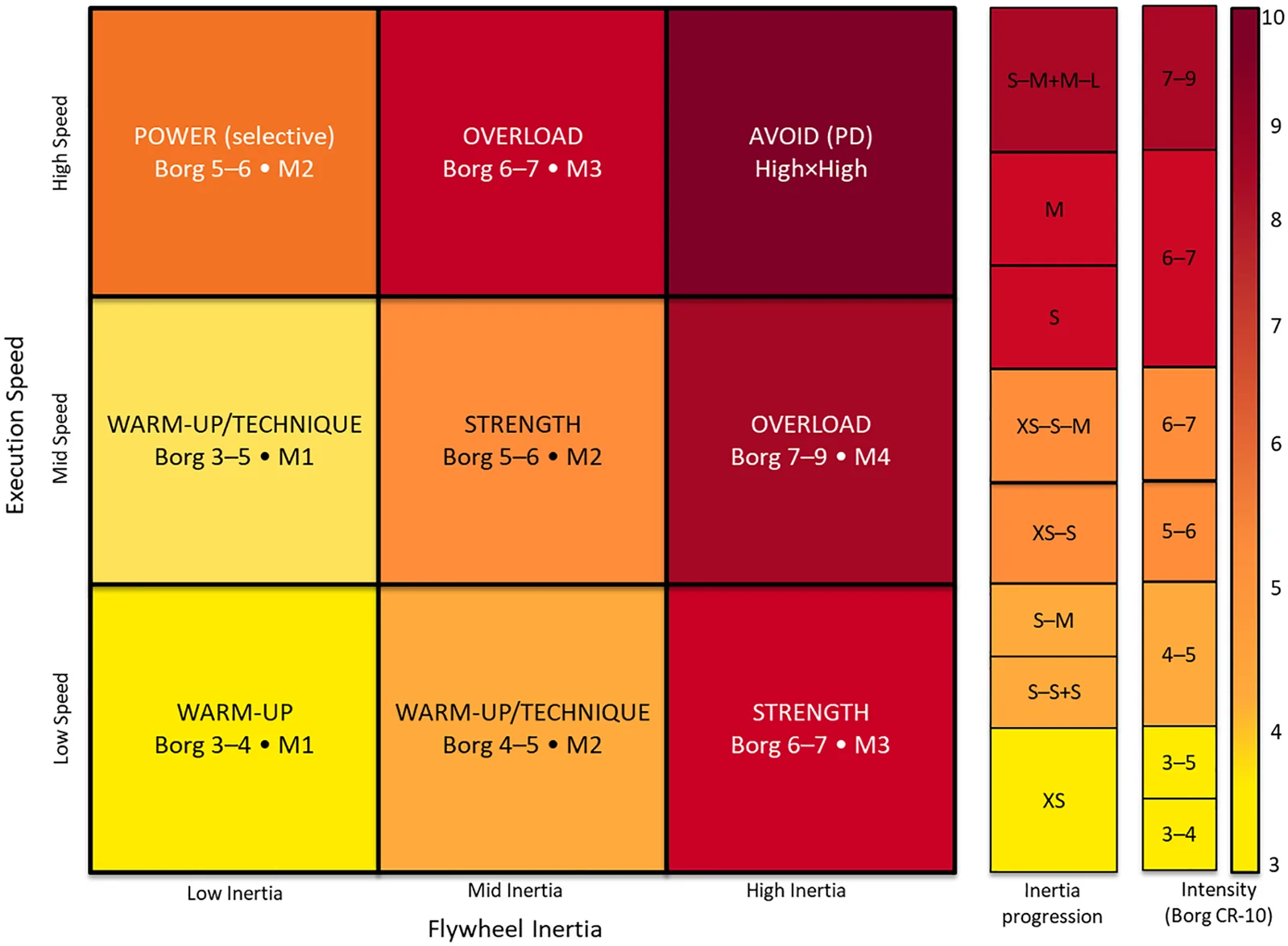
Flywheel training zones for PD in the PARKEX protocol. Zones were defined according to execution speed categories (low–mid–high) and flywheel inertia, with intensity progression guided by the Borg CR-10 scale throughout mesocycles.

Flywheel inertia (XS–L) and concentric movement velocity were adjusted to modulate eccentric overload while maintaining safety in people with PD. Mechanical variables displayed by the kMeter system (Exxentric AB, Stockholm, Sweden) were used as real-time biofeedback during exercise execution, allowing participants to visually associate their performance with perceived effort. Perceived exertion was assessed using the Borg CR-10 scale by asking: *How difficult was this combination of strength, speed, and inertia?*. These ratings were used to guide training progression across mesocycles. The load prescription strategy was further informed by the Flywheel Workout Zones, which maps training stimulus according to the interaction between inertial load and concentric speed [18,19]. For the PARKEX protocol, this framework was adapted to the specific neuromechanical requirements of PD, integrating Borg CR-10 intensity ranges and mesocycle progression (Fig 2). We linked inertia, intended speed, Borg targets and mesocycles as follows: technique and warm-up sessions used small (S) to medium (M) inertia (0.010–0.025 kg·m^2^) at low speed and Borg 3–5, typically during M1–M2; strength sessions used M inertia (0.025 kg·m^2^) at low-to-moderate speed and Borg 5–7, during M2–M3; power sessions used extra small (XS) to S inertia (0.005–0.010 kg·m^2^) at high speed with strict form, Borg 6–8, and short sets, typically in M2; and eccentric overload sessions used M–large (L) inertia (0.025–0.050 kg·m^2^) with maximal concentric intent and a prolonged eccentric phase, Borg 7–9, during M3–M4, avoiding the high-inertia × high-speed corner for safety in PD (Fig 2).

A summary of the 12-week training program can be seen in Table 1:

**Table 1:** Overview of the flywheel training program for PD.

Week	Mesocycles	Objectives	Training Zones	Inertia Progression According to the Borg Scale	Intensity (Borg CR-10)
0	Baseline	Initial assessment, technical introduction and load familiarization	Warm-up	–	–
1	Mesocycle I	Technique familiarization and load adaptation	Warm-up	XS	3-5
2			Warm-up	XS	3-5
3			Warm-up	S-S + S	4-5
4			Warm-up	S-M	4-5
5	Mesocycle II	Initial load progression and dual-task training initiation (BPT + FE group)	Technique	XS-S-M	5-7
6			Strength	XS-S-M	5-7
7	Monitor week	Load and technique adjustment (Borg+inertia)	Technique Strength	XS-S-S + S-M-S + M	4-7
8	Mesocycle III	Post-monitoring individualized progression	Strength	S	6-8
9				M	6-8
10				S-M	6-8
11	Mesocycle IV	Final intensification	Overload	S-M + M-L	7-9
12			–	S-M + M-L	7-9
Final week	Post-intervention	Post-intervention assessment	–	–	–

XS: extra small; S: small; M: medium; L: large.

Monitoring Week – Mid-intervention week dedicated to monitoring technical execution and perceived exertion using the Borg scale.

The Flywheel inertia disks: XS (0.005 kg·m^2^), S (0.010 kg·m^2^), M (0.025 kg·m^2^), L (0.050 kg·m^2^).

The BPT + FE group performed the same motor training as the BPT group, with the addition of concurrent executive-function tasks integrated directly into the exercises. Cognitive and motor demands were applied simultaneously to induce cognitive–motor interference. Dual-task elements included verbal fluency or structured verbal tasks during resistance exercises, reading modified or non-meaningful text aloud while performing motor tasks, ball-passing activities combined with lower-limb coordination tasks, and corridor-based gait training performed concurrently with cognitive challenges. A summary of the structure and progression of the dual-task training component can be seen in S1 Table.

Functional transfer was a key pillar of the program, integrating multi-joint exercises in functional positions (1-min Sit-to-Stand repetitions; STS), presses, rows, lunges) and complex combinations such as the row-squat, which simultaneously challenged strength, postural control, balance, and cardiovascular adjustment [18]. A demonstration of the eccentric flywheel exercises used in the program can be seen in S2 File. Sessions were conducted during the ON-medication phase when feasible; used harness/hand support as needed, and participants were instructed not to release the flywheel during the eccentric phase. To illustrate the cognitive-motor integration achieved during training, examples of the dual-task exercises performed by the patients are shown in S3 File and S4 File.

### Outcome measures

Clinical, motor, non-motor, and quality-of-life measures were prospectively collected as secondary outcomes within the PARKEX trial to explore the broader functional impact of the intervention. The primary endpoint of the overall trial was mitochondrial function, as described in the previously published study protocol. In the present analysis functional mobility measured by the Timed Up and Go (TUG) was considered the main clinical outcome. Additional outcomes included lower-limb muscular endurance (STS Test), health-related quality of life (PDQ-39 total and subscales), and depressive symptoms (BDI).

Assessments were conducted at baseline and after the 12-week intervention period. To account for potential confounding factors, four covariates were included in the analysis: sex, age, body mass index (BMI), and levodopa equivalent daily dose (LEDD). LEDD was calculated following the updated recommendations by Jost et al. (2023), which provide standardized proposals for dose equivalency in PD [20]. Motor symptom severity was assessed using the Unified Parkinson’s Disease Rating Scale Part III (UPDRS-III) [21]. Cognitive function was evaluated using the MoCA [22]. Non-motor symptoms were assessed with the Movement Disorder Society-Non-Motor Symptoms Scale (MDS-NMS) [23], and sleep disturbances were measured using the Parkinson’s Disease Sleep Scale (PDSS) [24].

The study examined four key dependent variables. Quality of life was assessed using the Parkinson’s Disease Questionnaire-39 (PDQ-39), a disease-specific instrument that evaluates eight dimensions relevant to PD, including mobility, emotional well-being, stigma, social support, cognition, communication, bodily discomfort, and activities of daily living [25,26]. Depressive symptoms were measured with the Beck Depression Inventory (BDI), a widely used 21-item self-report scale assessing the severity of depressive symptoms [27]. Overall satisfaction was evaluated using the Client Satisfaction Questionnaire (CSQ-8), an eight-item tool providing a global measure of satisfaction with health and social services [28]. Functional performance was evaluated through two physical assessments: the STS test, which measured lower-body muscular endurance by recording the maximum number of STS repetitions performed with proper technique [29], and the Timed Up and Go (TUG) test, which evaluated functional mobility by measuring the time taken to stand up from a chair, walk three meters, turn around, return, and sit down again [30,31].

### Statistical analysis

Descriptive statistics were computed for demographic and clinical variables across the three treatment groups. For each continuous variable (Age, BMI, Years since diagnosis, Hoehn & Yahr stage, UPDRS Total at T1, MoCA at T1, MDS-UPDRS Total at T1, and PDSS-2 Total at T1), we reported the median and interquartile range (IQR), as the distributions were non-normal. To assess differences between groups, we employed non-parametric Kruskal–Wallis tests for each continuous variable. The categorical variable Sex was analyzed separately using a chi-squared test of independence. Data analysis was performed using the R programming language v.4.3.1 on the RStudio integrated development environment (IDE) v.2023.9.1.494 [32,33].

A permutation-based analysis of variance (permutation ANOVA, aovp) was conducted to examine the effect of study group on the outcome variables (BDI, PDQ-39, STS, and TUG), while controlling for sex, age, BMI, and UPDRS-III score as covariates. UPDRS-III was selected instead of UPDRS total score because it provides a specific and validated measure of motor severity, directly linked to functional mobility and strength outcomes. The total score combines motor and non-motor domains, which may reflect broader disease burden rather than motor severity per se.

Analyses were performed with 200,000 permutations and a fixed random seed to ensure stable and reproducible p-value estimates. This method was selected for its robustness against violations of normality and homoscedasticity assumptions, making it particularly suitable for small sample sizes. To further explore the results obtained in the permutation ANOVA, pairwise permutation tests were conducted with Bonferroni correction to adjust for multiple comparisons. These tests assessed differences between study groups for all the outcome variables. For each pairwise comparison, the permutation-based test statistic was calculated along with the Bonferroni adjusted p-value. Median differences between groups were computed in each iteration, as medians are more robust than means against non-normal distributions and outliers, ensuring a more reliable estimation of central tendency in skewed data. Bootstrapped 95% confidence intervals were then estimated using 1000 resamples, with the 2.5 and 97.5 percentiles defining the interval. The rank-biserial correlation (r_rb) was used to measure effect size. This approach provides a robust estimation of group differences without relying on parametric assumptions.

Missing data was handled using mixed-effects models that accommodate missing values under the missing-at-random assumption. Sensitivity analyses were performed using multiple imputation to assess the robustness of results.

### Ethical considerations

The current clinical study has received approval from the Research Ethics Committee of the Faculty of Psychology and Education and Sports Sciences (Blanquerna, Universitat Ramon Llull) on 27/01/23 (2021008D), as well as from the Ethics Committee for Research with Medicines at Vall d’Hebron University Hospital (PR(AG)574/2021).

## Results

### Baseline clinical and demographic characteristics

A total of 24 individuals diagnosed with early-stage PD were randomly allocated to three groups: BPT, BPT + FE, and Con (Fig 1). Baseline, demographic and clinical characteristics are summarized in Table 2.

**Table 2:** Baseline, demographic, and clinical characteristics by study group.

Variable	Con	BPT	BPT + FE	*p*-value
Sex (M/F), number	2/ 5	3/ 3	6/ 2	.258
Age (years)	66.5 (7.9)	63.3 (6.9)	59.5 (10.1)	.197
BMI (kg/m²)	32.0 (4.0)	23.2 (7.1)	22.5 (3.8)	.0165*
Disease duration (years)	2.0 (2.0)	3.5 (2.5)	6.0 (3.8)	.273
Hoehn & Yahr Stage	2.0 (0.0)	2.0 (0.0)	2.0 (0.0)	.652
UPDRS total	51.0 (12.0)	34.5 (11.0)	39.0 (13.0)	.0339*
MoCA scale	26.0 (1.5)	30.0 (0.8)	28.0 (2.3)	.00846*
MDS-NMS	10.0 (10.5)	8.0 (8.0)	19.5 (5.0)	.0374*
PDSS	0.73 (0.47)	0.73 (0.40)	0.57 (0.37)	.542
ΔLEDD	−0.65	−1.04	0	.334

M: Male; F: Female, BMI: Body Mass Index; UPDRS: Unified Parkinson’s Disease Rating Scale; MoCA: Montreal Cognitive Assessment; MDS-NMS: Movement Disorder Society Nonmotor Rating Scale; PDSS: Parkinson’s Disease Sleep Scale; Data are presented as median (Interquartile range); ΔLEDD: Difference in levodopa equivalent daily dose (pre- vs. post-intervention).

The sample consisted of 24 participants, all classified as Hoehn & Yahr stage II, reflecting a clinically homogeneous group with mild-to-moderate disease severity. This homogeneity ensured minimal intergroup variability, thereby reinforcing both the internal validity and clinical relevance of the study findings. Of note, the 12-week physical intervention showed no consistent effect on reducing antiparkinsonian medication (LEDD), which remained stable throughout the study and therefore was not considered an influencing variable.

No intervention-related adverse events were observed, and only one participant discontinued the intervention due to physical discomfort unrelated to the physical activity program.

### Impact of training programs on physical performance

**Fig 3:**
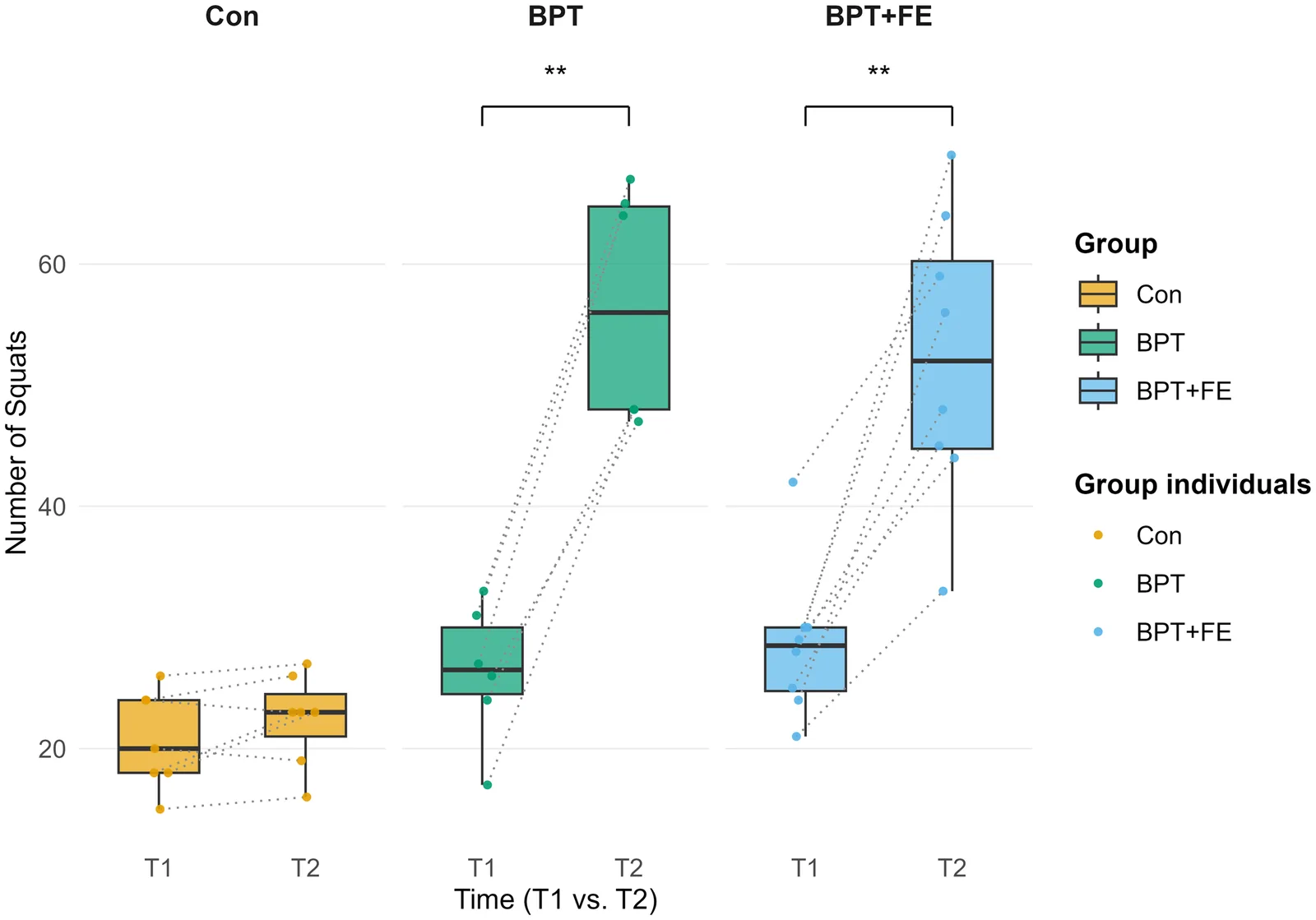
Number of STS repetitions completed pre- and post-intervention across the three groups. Colored bars represent pre- and post-intervention values; grey dotted lines unite individual scores for T1 and T2. *p* < .05 (*), p < .01 (**), p < .001(***).

A highly significant effect of the group on lower limb strength, as measured by the 1-min STS Test was observed (p = .001), indicating that the intervention significantly improved this outcome (Fig 3, Table 3). None of the covariates (sex, age, BMI or UPDRS-III motor scale) exhibited a significant association with STS Test performance (p-values ranging from.09 to 1).

**Table 3:** Permutation ANOVA results for group differences in quality of life (PDQ-39), depression (BDI), and physical function (1-min STS, TUG) measures.

Variable	Factor	*F*-statistic	*p*-value
PDQ-39	Group	0.51	.696
BDI	Group	1.41	.346
1-min STS	Group	19.11	.001**
TUG	Group	15.10	.004**

PDQ-39: Parkinson’s Disease Questionnaire; BDI: Beck’s Depression Inventory; STS: Sit-to-Stand; TUG: Timed-up and go. Note. *p*-values from permutation ANOVA (*aovp*). *p* < .05 (*), p < .01 (**), p < .001(***).

Post-hoc pairwise permutation tests revealed statistically significant differences between BPT and Con (p < .003), and between BPT + FE and Con (p < .003), but not between BPT and BPT + FE (p = .1854). The median difference between BPT and Con was 31.00 repetitions (r_rb = 1.000), and 19.00 between BPT + FE and Con (r_rb = 1.000), indicating strong group effects on both interventions on lower limb strength (Table 4).

**Table 4:** Pairwise comparisons of group differences on depression, quality of life, and physical performance measures. Comparison results including effect sizes, confidence intervals, and adjusted significance levels.

Comparison	Z-statistic	MD	IC (95%)	r_rb	Adjusted *p*-value
**BDI**					
BPT vs. BPT + FE	1.247	0.50	[-1.50, 6.00]	0.417	0.3186
BPT vs. Con	−0.9438	−0.50	[-13.00, 4.50]	−0.190	0.3453
BPT + FE vs. Con	−1.456	−1.00	[-15.00, 1.50]	−0.429	0.3186
**PDQ-39**					
BPT vs. BPT + FE	0.7279	4.50	[-9.50, 13.50]	0.188	0.5178
BPT vs. Con	−0.6467	−2.00	[-17.00, 10.50]	−0.214	0.5178
BPT + FE vs. Con	−1.182	−6.50	[-22.51, 7.02]	−0.339	0.5178
**STS**					
BPT vs. BPT + FE	1.324	12.00	[-6.00, 18.01]	0.417	0.1854
BPT vs. Con	3.313	31.00	[21.00, 36.00]	1.000	.0026 **
BPT + FE vs. Con	3.129	19.00	[12.00, 33.02]	1.000	.0026 **
**TUG**					
BPT vs. BPT + FE	0.717	0.37	[-0.69, 1.15]	0.208	0.4737
BPT vs. Con	−3.015	−2.07	[-3.13, -1.28]	−1.000	.0039 **
BPT + FE vs. Con	−3.308	−2.43	[-3.46, -1.48]	−1.000	.0028 **

PDQ-39: Parkinson’s Disease Questionnaire; BDI: Beck’s Depression Inventory; STS: Sit-to-Stand; TUG: Timed-up and go; MD = Median differences. BPT: Basic functional training, BPT + FE: BPT+Functional exercises; Con: control. Note. Pairwise permutation tests with Bonferroni correction for multiple comparisons. *p* < .05 (*), p < .01 (**), p < .001 (***).

**Fig 4:**
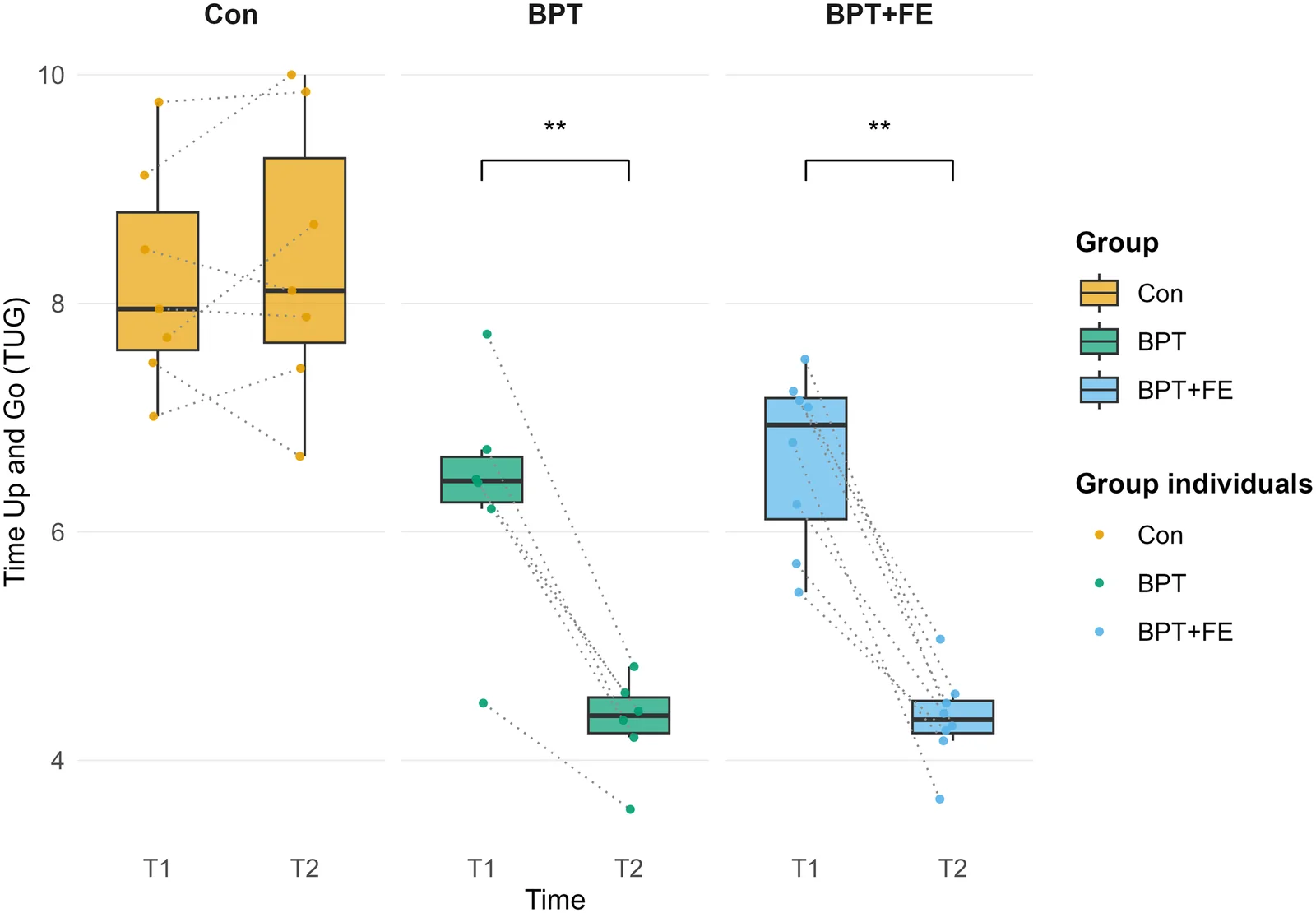
Time Up and Go (TUG) performance in seconds before and after the intervention for each group. Colored bars indicate pre- and post-intervention scores; grey dotted lines unite individual scores for T1 and T2. Lower values indicate better mobility. *p* < .05 (*), p < .01 (**), p < .001(***).

A significant effect was also found for the TUG test (p = .0042), reflecting group-related differences in functional mobility. None of the covariates (sex, age, BMI, or UPDRS-III motor scale) exhibited a significant association (p > .10 for all), suggesting that TUG performance was primarily influenced by the intervention type (Fig 4, Table 3).

Pairwise permutation tests indicated significant differences between BPT and Con (p = .0039; MD = −2.07; r_rb = −1.000) and BPT + FE and Con (p = .0028; MD = −2.43 seconds; r_rb = −1.000), but no significant difference between BPT and BPT + FE (p = .4737, Table 4).

### Effects of exercise on overall satisfaction, quality of life and cognitive function

The CSQ-8 results demonstrated high levels of satisfaction, with mean scores ranging from 3.4 to 4.0 indicating overall positive feedback.

**Fig 5:**
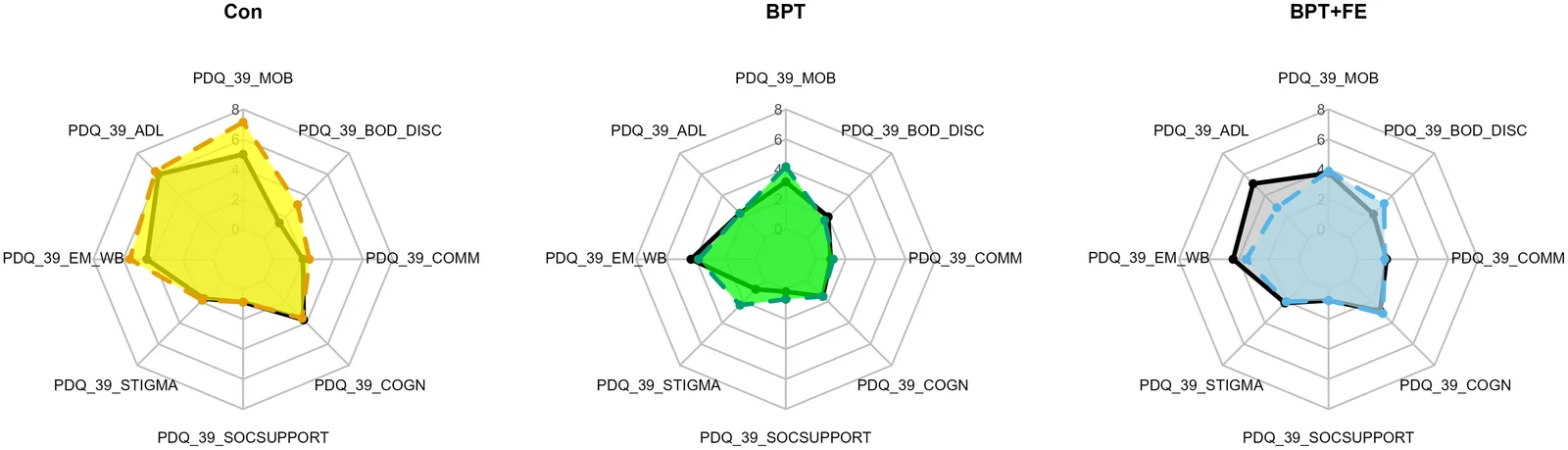
Radar plot representing pre- and post-intervention scores on the eight dimensions of the PDQ-39 for each group. Colored-dashed lines indicate post-intervention scores, while black-and-white lines represent baseline values. Lower scores indicate better perceived health-related quality of life across all subscales.

The permutation ANOVA revealed no significant overall effect of the intervention groups on PDQ-39 (p = .696) (Fig 5, Table 3). Likewise, none of the covariates (sex, age, BMI, or UPDRS-III motor score) showed a significant association with quality-of-life outcomes (p > .6 for all). Nevertheless, some PDQ-39 subscales presented non-statistically significant (p > .2 for all) medium to large effect sizes: in the BPT group versus control, Emotional Well-Being (MD = −3.00, IC95 = [−5.00, 1.00]) and Bodily Discomfort (MD = −0.50, IC95 = [−4.50, 1.00]) reached r_rb = −0.428 and −0.524, respectively, while in the BPT + FE group versus control, Activities of Daily Living (MD = −1.50, IC95 = [−8.00, 1.01]) and Emotional well-being (MD = −3.00, IC95 = [−5.50, 0.50]) showed r_rb = −0.429 and −0.446 (Fig 5).

Pairwise comparisons also indicated no statistically significant differences between groups (p > .51 for all). However, the median difference between BPT + FE and Con was −6.50 (r_rb = −0.339), suggesting a small to moderate trend toward improved quality of life in the BPT + FE group. Although not statistically significant, the observed pattern may reflect a clinically meaningful effect that merits further exploration. The other comparisons showed smaller median differences (MD < 4.60, |r_rb| < 0.22), suggesting a minimal variation across groups (Table 4).

No significant between-group differences were observed in global cognitive function (MoCA).

### Effects of exercise on depressive symptoms

The intervention groups did not have a statistically significant effect on depressive symptoms as measured by the BDI, with the overall permutation ANOVA yielding *p* = .577. None of the covariates (sex, age, BMI or UPDRS-III motor score) exhibited a significant association with BDI scores (p-values ranging from.27 to.84) (Table 3). However, post-hoc comparisons showed a median difference of −1.00 (r_rb = −0.429) between BPT + FE and Con, suggesting a possible moderate reduction in depressive symptoms in the BPT + FE group. The differences between the other group comparisons were smaller (MD < 0.60, |r_rb| < 0.42), indicating that any overall group effects on depressive symptoms were likely limited (Table 4).

## Discussion

This study evaluated the clinical and functional outcomes of two exercise interventions (BPT and BPT + FE) in individuals with early-stage PD, compared to usual care (no intervention). Although no PDQ-39 subscales reached statistical significance, medium effect-size trends were observed for activities of daily living and emotional well-being in the BPT + FE group. Similarly, in the BPT group, medium-to-large effect-size patterns were also observed for Emotional Well-Being and Bodily Discomfort, suggesting potential improvements in these domains despite the absence of statistical significance. Both intervention groups showed statistically significant and clinically meaningful improvements in physical performance, with large effects on lower-limb strength (1-min STS Test) and functional mobility (TUG test). These findings highlight the robust impact of structured exercise interventions on motor function and support the integration of both basic and dual-task training in early PD management.

Exercise is increasingly recognized as an important component of PD management, supporting its integration into standard care for symptomatic relief and for its potential disease-modifying effects [34]. For example, regular PA is associated with a reduced risk of developing PD, with prospective cohort studies suggesting approximately 20–30% lower incidence among those engaging in higher levels of exercise [35]. Similarly, clinical trials have begun to test whether exercise can slow disease progression. In this line, the SPARX3 phase III multicenter trial is evaluating the impact of moderate versus high-intensity aerobic exercise on motor symptoms in early PD patients [36], while a phase II randomized trial showed that high-intensity treadmill training was associated with attenuated progression of motor symptoms in patients with de novo PD [37]. These findings align with other clinical trials and reviews, such as the Park-in-Shape study which demonstrated positive effects of aerobic or multimodal training on motor outcomes, though the impact on non-motor symptoms remains uncertain [38]. However, the heterogeneity in outcomes across studies underscores the need for more tailored, person-centered approaches that consider cognitive engagement, emotional factors, and adherence. Emerging evidence suggests that dual-task functional exercises may confer greater benefits than single-task training by promoting neuroplastic adaptations in both the general population and individuals with PD [10,11]. In healthy adults, dual-task training enhances connectivity and efficiency in motor-cognitive neural networks, specifically in the prefrontal cortex, anterior cingulate cortex, and basal ganglia, leading to improved automaticity in movements and executive functioning. Moreover, dual-task gait training has been shown to promote functional reorganization in brain networks associated with executive-attentive control and motor coordination, supporting improved cognitive–motor integration in PD patients [39,40].

In both intervention programs (BPT and BPT + FE), the core strength–endurance component was performed using eccentric resistance training. This methodological choice was selected based on safety, efficacy, and personalization criteria, particularly relevant for people with PD. The training protocol used the Flywheel kBox4 device, due to its ability to generate a natural eccentric overload, made possible because kinetic energy accumulated during the concentric phase is released upon reversal without the need for manipulating external weights, thereby enabling high neuromuscular activation even in populations with motor impairments such as PD [17]. This choice was also supported by the physical operating principle of flywheel devices, where the load is entirely inertial; therefore, regardless of how large the inertia and how small the exerted force, the flywheel can always rotate [41]. A distinctive feature of moderate-to-high load eccentric exercise is its markedly reduced metabolic cost [42], which can be up to four times lower than that of concentric exercise at equivalent mechanical loads [42,43]. This characteristic is particularly relevant for individuals with PD, who often exhibit central fatigue, reduced energy reserves, and proximal muscle weakness [44]. In fact, eccentric modalities may be ideally suited for subjects with PD because high levels of muscle force are generated with low metabolic demands [42,45]. The observed gains in physical performance are consistent with prior literature suggesting that structured exercise can enhance strength and mobility in individuals with PD [46,47].

Importantly, the BPT + FE group was designed as a true dual-task condition, integrating concurrent cognitive and motor demands rather than adding sequential cognitive exercises. Notably, both intervention types led to improvements over the control group, with no significant differences between BPT and BPT + FE. The overall PDQ-39 and BDI scores showed medium effect sizes, suggesting potentially meaningful benefits in quality of life and mood. Although the overall analysis did not reveal significant effects on quality of life (PDQ-39), further inspection of the subscales suggested clinically relevant patterns. Medium-to-large effect sizes were observed in several domains: in the BPT group compared with controls, improvements were noted in emotional well-being and bodily discomfort, while in the BPT + FE group, favorable effects emerged for activities of daily living and emotional well-being. These findings, although not statistically significant, point to meaningful improvements in patient-perceived outcomes that may require larger cohorts or longer interventions to reach conventional significance thresholds. Importantly, such changes in emotional and daily functioning dimensions align with the broader literature emphasizing the psychosocial impact of exercise in PD, and underscore the potential added value of integrating structured training into routine care [46].

Importantly, dual-task training has been shown to produce greater improvements in gait speed, stride length, and balance, compared to single-task approaches. A recent meta-analysis of 17 RCTs (826 participants) reported moderate to large standardized effect sizes for dual-task training improving walking speed (SMD 0.42), stride length (SMD 0.69), and balance (SMD 1.15) over single-task or usual care interventions [48]. Moreover, additional RCTs have found dual-task protocols to be particularly effective in enhancing functional walking velocity and postural control. However, as with our findings, the evidence on quality-of-life outcomes is mixed: some studies suggest a QoL benefit, while others do not [48]. Thus, our results, showing robust physical improvements with enhanced well-being in the BPT + FE group, contribute to a growing body of research advocating for cognitively integrated exercise programs in PD.

The study findings also align with existing evidence demonstrating that exercise interventions improve specific aspects of quality of life, such as activities of daily living. A recent meta-analysis confirmed that both aerobic and resistance training can yield significant benefits for mood and daily functioning [49]. These improvements are likely mediated by neuroplastic changes, enhanced self-efficacy, and social engagement during exercise sessions. The study also aligns with our findings, highlighting that specific motor symptoms may be treated most effectively by PD‐specific programs [49]. Similarly, structured exercise programs have also been shown to reduce depression in people with PD [50,51]. This suggests that performing continuous PA may represent an accessible, non-pharmacological strategy to improve patient well-being. It becomes relevant to conduct long-term studies to truly determine the effectiveness of BPT + FE interventions in improving individuals’ quality of life and delaying disease progression.

Our findings align with the evolving paradigm in PD care that advocates for integrative, non-pharmacological strategies aimed at both symptom control and disease modification. As highlighted by Bloem and colleagues, future care models must move beyond motor-centric approaches to embrace interventions that address the full spectrum of physical, cognitive, and emotional needs [52]. In this context, we propose the concept of vital holism—a comprehensive therapeutic orientation that recognizes the dynamic interplay between health, neuroplasticity, psychological resilience, and lived experience. This framework supports the idea that exercise, when designed holistically, becomes not merely a physical intervention, but a systemic modulator of quality of life and biological function in PD. Furthermore, our perspective integrates the emerging model of neural-systemic dual plasticity, which describes coordinated plastic adaptations in both the central nervous system and peripheral bioenergetic systems [53]. We also expand upon the distinction between primary and secondary plasticity, as outlined in the PARKEX protocol, where exercise-induced mitochondrial remodeling is posited as a foundational mechanism underlying sustained clinical improvements [16]. Our results support this integrative perspective, particularly as both physical and cognitive aspects of exercise were associated with enhanced mobility and strength.

### Limitations

Importantly, the study was powered for the primary mitochondrial endpoint and not for clinical scales. Therefore, the absence of significant differences in some outcomes may reflect limited statistical power rather than true absence of effect. Baseline imbalance across key variables (BMI, MoCA, UPDRS, MDS-NMS) limits internal validity and may have influenced the observed effects despite adjustment. The small sample size further limits statistical power and generalizability, and results should therefore be interpreted with caution. The short duration of the intervention (12 weeks) may not be sufficient to capture long-term effects, especially on quality of life and depressive symptoms. Moreover, the limited timeframe may also explain the absence of observable changes in patients’ LEDD. Longer-term studies, such as the 3.5-year Tai Chi follow-up demonstrating beneficial effects on PD, with an improvement in motor and non-motor symptoms and reduced complications, highlight the need for extended intervention periods to fully understand sustained benefits [54]. It is worth noting that muscle strength is known to be influenced by sex. While the distribution of sex was balanced across groups, this factor may still play a role and warrants consideration when interpreting the results. Blinding of participants was not feasible due to the nature of the interventions, which could introduce expectancy biases. In addition, the inclusion of a usual-care control group without an attention-matched intervention may have further increased the risk of expectation and attention bias. This factor may be particularly relevant for self-reported outcomes (PDQ-39, BDI) and effort-dependent measures such as the TUG. Additionally, the TUG was assessed only under single-task conditions. The inclusion of dual-task conditions, integrating simultaneous motor and cognitive demands, could have provided additional insights into the effects of the intervention under more complex functional situations. Given our results, future research should continue to explore optimal exercise modalities and intensities to maximize all physical, functional and psychosocial benefits, especially in early-stage patients where quality-of-life preservation is a key management goal.

## Conclusion

In summary, the PARKEX trial demonstrates that both BPT and dual-task exercise (BPT + FE) interventions can produce meaningful improvements in physical function in early-stage PD. While patient-reported outcomes showed only trends, the magnitude of change in motor performance supports the integration of structured exercise, especially those incorporating strength and functional mobility, as a core component of early PD management. Moreover, the observed levels of patient satisfaction and adherence suggest that such exercise programs are feasible and acceptable, supporting their integration as an additional pillar of comprehensive PD treatment. Future research should explore longer-term effects, and the potential for tailoring interventions to individual profiles of motor and non-motor symptoms.

## Supporting information

S1 Table Structure and mesocycle-based progression of the dual-task training component implemented in the BPT + FE group. (DOCX)

S2 File Representative recording of a training session combining flywheel-based resistance exercises with corridor-based gait components. (MP4)

S3 File Ball-passing coordination dual-task exercise. Representative example of a motor dual-task exercise in which participants perform ball-passing activities while simultaneously executing lower-limb coordination tasks.
(MP4)

S4 File Cognitive–motor dual-task exercise. Representative example of a dual-task exercise in which participants read non-meaningful text aloud while performing a ball-passing motor task.
(MP4)
