# Supplementary material for: Effects of basic and dual-task training programs on physical function in Parkinson’s disease: The PARKEX study
Source: PLoS One. 2026 Sep 18;21(9):e0358534. doi: 10.1371/journal.pone.0358534 (PMC13588342; doi:10.1371/journal.pone.0358534)
Supplement: Supplementary file 1 [file pone.0358534.s001.docx]

S1 Table. Structure and mesocycle-based progression of the dual-task training component implemented in the BPT+FE group.

| **Week** | **Mesocycles** | **DTT** | **Motor–Motor (M–M)** | **Motor–Cognitive (M–C)** | **Session phase, frequency and duration** |
| --- | --- | --- | --- | --- | --- |
| 0–4 | Mesocycle I | No DTT | — | — | — |
| 5–6 | Mesocycle II | Initiation and early progression of DTT | Introduction of structured coordinative motor tasks with gradual increase in difficulty. Locomotor training with progressive speed increments (from slow walking to jogging or running over 15–20 meters) while performing simultaneous manipulative tasks (carrying objects, ball dribbling). Initially, individually and subsequently in pairs. | Introduction of structured cognitive tasks with gradual increase in difficulty. Locomotor training over a 15–20-meter distance while performing concurrent mental arithmetic tasks of increasing complexity (e.g., counting ascending even numbers, ascending odd numbers). | Performed in each session (3 sessions/week); implemented during warm-up, transitions/active rest, and cool-down phases; total duration per session: 12–15 minutes. |
| 7 | Monitor week | Consolidation of DTT | Maintenance of previously introduced motor–motor task complexity. Consolidation of tasks implemented during Mesocycle II. | Maintenance of previously introduced motor–cognitive task complexity. Consolidation of tasks implemented during Mesocycle II. |  |
| 8–10 | Mesocycle III | Progressive increase of DTT | Increased coordinative task difficulty. Mesocycle II activities with the addition of external obstacles (cones, hoops). Introduction of 3-participant weaving drills using a single shared ball (combined locomotor displacement and coordinated ball exchange), (see S3 Video). | Increased cognitive task difficulty. Counting ascending and descending even and odd numbers while negotiating obstacles; naming animals and countries. |  |
| 11–12 | Mesocycle IV | Final phase of DTT progression | Further progression of coordinative task difficulty. 3-participant weaving drills using a single shared ball, performed at higher speeds and requiring greater inter-participant coordination. | Further progression of cognitive task difficulty. Standing in a static position while performing graded manipulative tasks—initially transferring the ball from one hand to the other, then dribbling individually, followed by paired ball exchanges—while reading aloud a song in a non-familiar language synchronized with the original audio (see S4 Video). The task progressed to structured group ball passing (two rows of four participants), with the ball circulating in clockwise and counterclockwise directions while reading. Once successfully completed, a second ball was introduced, requiring continuous circulation without collision between balls while maintaining the reading task. |  |

Note: DTT = dual-task training; M–M = motor–motor; M–C = motor–cognitive
