# Supplementary material for: Effects of basic and dual-task training programs on physical function in Parkinson’s disease: The PARKEX study
Source: PLoS One. 2026 Sep 18;21(9):e0358534. doi: 10.1371/journal.pone.0358534 (PMC13588342; doi:10.1371/journal.pone.0358534)
Supplement: Supplementary file 2 [file pone.0358534.s005.docx]

Dear Editor,

I have reviewed the manuscript entitled “Effects of basic and dual-task training programs on physical function in Parkinson’s disease: the PARKEX study”.

The authors completed the drug therapy with physical training in Parkinson’s disease. The treated groups were compared with a group without physical training. They followed investigation lasted for 12 weeks. The training was applied three times a week. It was a progressive training.

The study is useful, because the effect of training has been shown different from that of drugs. The applied interesting equipment named; “ flywheel device” was used in the course of traditional gymnastic. The description of the training is difficult to understand; I recommend to rewrite it. How many patients with Hoehn-Yahr I, II, III were included in the treated groups?

What was the process of dual-task training? Was it applied in the same arrangement for 12 weeks? How many times was it applied?

You cited literature of dual-task training, but your results not confirmed them. There has been a publication on a dual-task train for 5 days (J Psychiatry Psychiatric Disord 2023). Can you discuss it comparing with your results?

I recommend the manuscript for major revision.
