# Supplementary material for: Effects of basic and dual-task training programs on physical function in Parkinson’s disease: The PARKEX study
Source: PLoS One. 2026 Sep 18;21(9):e0358534. doi: 10.1371/journal.pone.0358534 (PMC13588342; doi:10.1371/journal.pone.0358534)
Supplement: Supplementary file 3 [file pone.0358534.s007.docx]

Dear Editor and Reviewers,

Thank you very much for taking the time to review our manuscript entitled *“Effects of Basic and Dual-Task Training Programs on Physical Function in Parkinson’s Disease: Results from the PARKEX Randomized Trial”* (PONE-D-25-60916).

We really appreciate your thoughtful and constructive feedback, which has helped us improve the clarity and overall quality of the paper.

We have revised the manuscript accordingly and made several changes based on your suggestions. Below, each of your comments (in **bold**) is followed by our response. All updates have also been highlighted in the revised version of the manuscript.

The PARKEX study protocol was previously published in PLOS ONE following peer review and has been uploaded as a Related Manuscript. The current manuscript reports the trial outcomes and includes methodological descriptions that may overlap with the published protocol (e.g., study design and intervention details), as required for transparency and reproducibility. No results, analyses, figures, tables, or outcome data from this trial have been previously published elsewhere. The manuscript is original, has not been published in whole or in part, and is not under consideration by any other journal. Therefore, this submission does not constitute dual publication.

Sincerely,

Joel Montane

**Journal Requirements:**

**When submitting your revision, we need you to address these additional requirements.
1. Please ensure that your manuscript meets PLOS ONE's style requirements, including those for file naming. The PLOS ONE style templates can be found at**
[**https://journals.plos.org/plosone/s/file?id=wjVg/PLOSOne_formatting_sample_main_body.pdf**](https://www.google.com/url?q=https://urldefense.com/v3/__https://track.editorialmanager.com/CL0/https:*2F*2Fjournals.plos.org*2Fplosone*2Fs*2Ffile*3Fid%3DwjVg*2FPLOSOne_formatting_sample_main_body.pdf/1/010f019c45736664-288e4aa0-f7bf-4ad8-a115-feba11de77e7-000000/hLeD9U1O1t_X2LQ11qsFvNYOdJjRVlXAWG2sNYv2oQ8%3D249__;JSUlJSUlJQ!!Bl-CRKAlSonS!DF8ace6eQG20Knjq83t1ZXR_eMlUcupsBlAvcO782sGuU_I16gh_-mEwDyCPtpG6S65r7c0To_6c51-arrpxXn-b$&source=gmail-imap&ust=1771296977000000&usg=AOvVaw2vpbYSEsiSF6uNcv_ITHPD) **and** [**https://journals.plos.org/plosone/s/file?id=ba62/PLOSOne_formatting_sample_title_authors_affiliations.pdf**](https://www.google.com/url?q=https://urldefense.com/v3/__https://track.editorialmanager.com/CL0/https:*2F*2Fjournals.plos.org*2Fplosone*2Fs*2Ffile*3Fid%3Dba62*2FPLOSOne_formatting_sample_title_authors_affiliations.pdf/1/010f019c45736664-288e4aa0-f7bf-4ad8-a115-feba11de77e7-000000/g7RUMLE-q-0TGrWU_xHjf7ywPHabd87GbuOyluFR_LI%3D249__;JSUlJSUlJQ!!Bl-CRKAlSonS!DF8ace6eQG20Knjq83t1ZXR_eMlUcupsBlAvcO782sGuU_I16gh_-mEwDyCPtpG6S65r7c0To_6c51-ariCeMftL$&source=gmail-imap&ust=1771296977000000&usg=AOvVaw3aLequu5utT8syud0FzXNq)

Response: We have carefully revised the manuscript to ensure full compliance with PLOS ONE style requirements.

**2. Your ethics statement should only appear in the Methods section of your manuscript. If your ethics statement is written in any section besides the Methods, please move it to the Methods section and delete it from any other section. Please ensure that your ethics statement is included in your manuscript, as the ethics statement entered into the online submission form will not be published alongside your manuscript.**

 Response: The ethics statement has been moved to the Methods section

**3. We noted in your submission details that a portion of your manuscript may have been presented or published elsewhere. A protocol for this clinical trial (PARKEX) was previously published in PLOS ONE. The current submission reports the trial outcomes and therefore includes brief methodological descriptions that may naturally overlap with the protocol (e.g., study design, participant eligibility criteria, and planned intervention components). These elements are required to ensure transparency and reproducibility and do not constitute duplicate publication.
No results, analyses, figures, tables, or outcome data have been published in any form before.

A copy of the published protocol has been uploaded as a Related Manuscript, as requested.**

**Please clarify whether this [conference proceeding or publication] was peer-reviewed and formally published. If this work was previously peer-reviewed and published, in the cover letter please provide the reason that this work does not constitute dual publication and should be included in the current manuscript.**

Response: The PARKEX study protocol was previously published in PLOS ONE following peer review and has been uploaded as a Related Manuscript. The current manuscript reports the trial outcomes and includes methodological descriptions that may overlap with the published protocol. No results, analyses, figures, tables, or outcome data from this trial have been previously published elsewhere. Therefore, this manuscript does not constitute dual publication. This has been explained in the cover letter above.

**4. In the online submission form, you indicated that the data supporting the findings of this study are not publicly available but can be obtained from the corresponding author upon request.
All PLOS journals now require all data underlying the findings described in their manuscript to be freely available to other researchers, either 1. In a public repository, 2. Within the manuscript itself, or 3. Uploaded as supplementary information.
This policy applies to all data except where public deposition would breach compliance with the protocol approved by your research ethics board. If your data cannot be made publicly available for ethical or legal reasons (e.g., public availability would compromise patient privacy), please explain your reasons on resubmission and your exemption request will be escalated for approval.**

Response: In accordance with the PLOS data availability policy, we have now deposited the dataset underlying the findings of this study in a public repository (Zenodo). The dataset is openly available under a Creative Commons Attribution 4.0 International (CC BY 4.0) license. The permanent DOI has been included in the revised Data Availability Statement in the manuscript.

**5. We note that Figure 1 in your submission contain copyrighted images. All PLOS content is published under the Creative Commons Attribution License (CC BY 4.0), which means that the manuscript, images, and Supporting Information files will be freely available online, and any third party is permitted to access, download, copy, distribute, and use these materials in any way, even commercially, with proper attribution. For more information, see our copyright guidelines:** [**http://journals.plos.org/plosone/s/licenses-and-copyright**](https://www.google.com/url?q=https://urldefense.com/v3/__http://journals.plos.org/plosone/s/licenses-and-copyright__;!!Bl-CRKAlSonS!DF8ace6eQG20Knjq83t1ZXR_eMlUcupsBlAvcO782sGuU_I16gh_-mEwDyCPtpG6S65r7c0To_6c51-arpPP0_C9$&source=gmail-imap&ust=1771296977000000&usg=AOvVaw136ONVPdVlJAuUcHbvAuzn)**.

We require you to either (1) present written permission from the copyright holder to publish these figures specifically under the CC BY 4.0 license, or (2) remove the figures from your submission:

a. You may seek permission from the original copyright holder of Figure 1 to publish the content specifically under the CC BY 4.0 license.

We recommend that you contact the original copyright holder with the Content Permission Form (**[**http://journals.plos.org/plosone/s/file?id=7c09/content-permission-form.pdf**](https://www.google.com/url?q=https://urldefense.com/v3/__http://journals.plos.org/plosone/s/file?id%3D7c09*content-permission-form.pdf__;Lw!!Bl-CRKAlSonS!DF8ace6eQG20Knjq83t1ZXR_eMlUcupsBlAvcO782sGuU_I16gh_-mEwDyCPtpG6S65r7c0To_6c51-arsB5yuqr$&source=gmail-imap&ust=1771296977000000&usg=AOvVaw1Uy0y7D45mNKlYAl_OoRi4)**) and the following text:
“I request permission for the open-access journal PLOS ONE to publish XXX under the Creative Commons Attribution License (CCAL) CC BY 4.0 (**[**http://creativecommons.org/licenses/by/4.0/**](https://www.google.com/url?q=https://urldefense.com/v3/__http://creativecommons.org/licenses/by/4.0/__;!!Bl-CRKAlSonS!DF8ace6eQG20Knjq83t1ZXR_eMlUcupsBlAvcO782sGuU_I16gh_-mEwDyCPtpG6S65r7c0To_6c51-armFQE9Mb$&source=gmail-imap&ust=1771296977000000&usg=AOvVaw1F1RWavmdYyAeSiBCfh4Iw)**). Please be aware that this license allows unrestricted use and distribution, even commercially, by third parties. Please reply and provide explicit written permission to publish XXX under a CC BY license and complete the attached form.”

Please upload the completed Content Permission Form or other proof of granted permissions as an "Other" file with your submission.

In the figure caption of the copyrighted figure, please include the following text: “Reprinted from [ref] under a CC BY license, with permission from [name of publisher], original copyright [original copyright year].”

b. If you are unable to obtain permission from the original copyright holder to publish these figures under the CC BY 4.0 license or if the copyright holder’s requirements are incompatible with the CC BY 4.0 license, please either i) remove the figure or ii) supply a replacement figure that complies with the CC BY 4.0 license. Please check copyright information on all replacement figures and update the figure caption with source information. If applicable, please specify in the figure caption text when a figure is similar but not identical to the original image and is therefore for illustrative purposes only.**

Response: Figure 2 has been completely redesigned and replaced with an original conceptual framework developed specifically for the PARKEX protocol. This new figure does not reproduce, adapt, or modify any previously published or copyrighted material and fully complies with the Creative Commons Attribution (CC BY 4.0) license requirements of PLOS ONE.

**6. We note you have included a table to which you do not refer in the text of your manuscript. Please ensure that you refer to Table 1 in your text; if accepted, production will need this reference to link the reader to the Table.**

 Response: Table 1 is explicitly referenced in the main text.

**7.** [**Please include captions for your Supporting Information files at the end of your manuscript, and update any in-text citations to match accordingly. Please see our Supporting Information guidelines for more information: http://journals.plos.org/plosone/s/supporting-information.**](https://www.google.com/url?q=https://urldefense.com/v3/__http://track.editorialmanager.com/CL0/http:*2F*2Fjournals.plos.org*2Fplosone*2Fs*2Fsupporting-information./1/010f019c45736664-288e4aa0-f7bf-4ad8-a115-feba11de77e7-000000/_bGgA5Z-FBkshIWQnTFbVcXRvL_wb1iRJNN5Pc9gKPM%3D249__;JSUlJSU!!Bl-CRKAlSonS!DF8ace6eQG20Knjq83t1ZXR_eMlUcupsBlAvcO782sGuU_I16gh_-mEwDyCPtpG6S65r7c0To_6c51-arj1UZ4Vu$&source=gmail-imap&ust=1771296977000000&usg=AOvVaw1nhGXF3gLZ8JvGIhMjuIJc)

Response: Captions for all Supporting Information files have been added at a specific section at the end of the manuscript to ensure consistency with PLOS ONE guidelines.

**8. If the reviewer comments include a recommendation to cite specific previously published works, please review and evaluate these publications to determine whether they are relevant and should be cited. There is no requirement to cite these works unless the editor has indicated otherwise.**

Response: We have carefully reviewed the publications suggested by the reviewers and have incorporated the relevant reference into the revised manuscript where appropriate.

**Reviewers' comments:**

**Reviewer #1: 1. Lines 148-151 (Sample Size) and Lines 229–231 (Outcomes): The N=24 calculation is based on a protocol for mitochondrial function, yet this paper reports only clinical outcomes like TUG and PDQ-39.
- Why is the original primary endpoint (mitochondrial function) omitted?
- Since the study was not powered for clinical scales, please provide a statistical justification for N=24 or reframe these results as exploratory secondary analyses.**

Response: Thank you for this important comment. The original sample size calculation (n = 24) was based on the primary endpoint of the PARKEX trial, the mitochondrial function, as described in the published study protocol. The present manuscript reports a pre-specified secondary analysis focusing on clinical, motor, non-motor, and quality-of-life outcomes collected within the same randomized controlled trial. While these clinical outcomes were prospectively assessed, the study was not specifically powered to detect clinically meaningful differences in these scales. Therefore, the findings should be considered exploratory in nature. We have clarified this important point in the Introduction, Methods (sample size section), and Limitations to improve transparency and avoid overinterpretation of the clinical results.

In the Introduction section:

“The PARKEX trial was originally designed to evaluate changes in mitochondrial function as its primary biological endpoint. In addition to these mechanistic outcomes, clinical, motor, non-motor, and quality-of-life measures were prospectively collected to explore the potential functional impact of the intervention.”

In the Methods section:

“The sample size (N=24) was calculated based on the primary mitochondrial endpoint of the trial, as detailed in the published protocol. Clinical and functional outcomes analyzed in the present manuscript were secondary endpoints and the study was not specifically powered to detect changes in these measures.”

“Clinical, motor, non-motor, and quality-of-life measures were prospectively collected as secondary outcomes within the PARKEX trial to explore the broader functional impact of the intervention. The primary endpoint of the overall trial was mitochondrial function, as described in the published protocol. In the present analysis, functional mobility measured by the Timed Up and Go (TUG) was considered the main clinical outcome. Additional outcomes included lower-limb muscular endurance (Squat Test), health-related quality of life (PDQ-39 total and subscales), and depressive symptoms (BDI).”

In the Limitations section:

“Importantly, the study was powered for the primary mitochondrial endpoint and not for clinical scales. Therefore, the absence of significant differences in some outcomes may reflect limited statistical power rather than true absence of effect.”

**2. Lines 369-377 (Results) and Lines 59–63 (Abstract): The authors report medium-to-large effect sizes for specific PDQ-39 subscales (Emotional Well-Being, Bodily Discomfort, Activities of Daily Living) with r_rb values ranging from –0.428 to –0.524. However, these subscale comparisons are not shown in Table 4 or any supplementary table. Table
4 only includes the total PDQ-39 score comparisons. Reporting specific numerical results for subscales without providing the accompanying statistical tests (MD, 95% CI, p-value, r_rb) violates CONSORT guidelines for complete outcome reporting and prevents readers from verifying these claims.**

Response: To address this concern and ensure complete outcome reporting, we have revised the manuscript to explicitly include, within the results main text, the corresponding mean differences (MD), 95% confidence intervals (95% CI), and p-values for the referenced PDQ-39 subscales.

**3. Lines 299-304 (Table 2): The legend uses "Median ± IQR." The "±" symbol is reserved for Mean ± SD. Please update the table to standard non-parametric notation: Median (IQR) or Median [Q1, Q3].**

Response: The table has been revised accordingly. The notation “Median ± IQR” has been replaced with the standard non-parametric format “Median (IQR)” throughout Table 2 to ensure correct statistical reporting.

**4. Lines 336-340 and Table 4: (Pairwise comparisons): While the permutation tests are appropriate for the small sample, the comparison between BPT and BPT+FE (n=8 per group) is severely underpowered to detect clinically meaningful differences between these two exercise modalities. The non-significant p-values (p > 0.1854 for all outcomes) should not be interpreted as evidence of equivalence or absence of added benefit from dual-task training. Any conclusions regarding the comparative efficacy of BPT versus BPT+FE should be explicitly tempered by this limitation.**

Response: We thank the reviewer for this important methodological consideration. We agree that the sample size limits the statistical power to detect clinically meaningful differences between the BPT and BPT+FE groups, and that non-significant results should not be interpreted as evidence of equivalence. Accordingly, we have removed the sections of the manuscript where conclusions were drawn regarding the comparative efficacy between BPT and BPT+FE under these conditions. The revised manuscript now avoids any interpretation suggesting equivalence or absence of added benefit and more appropriately reflects the exploratory nature of these comparisons given the limited sample size.

**5. Lines 124-128 (Study Design): The use of a no-intervention control group (the 3rd arm) introduces significant risk of expectation and attention bias. This is a major limitation for self-reported (PDQ-39, BDI) and effort-dependent (TUG) outcomes. Please discuss how this bias was addressed or acknowledge it as a factor that may inflate the observed benefits.**

Response: We agree that the use of a no-intervention control group may introduce expectation and attention bias, particularly for self-reported and effort-dependent outcomes. The choice of this design was based on the aim to evaluate the added effect of structured exercise compared with usual care. However, we acknowledge that this approach may inflate perceived benefits.

We have now addressed this issue in the Limitations section, clarifying the potential influence of expectation and attention bias on PDQ-39, BDI, and TUG outcomes, and cautioning against overinterpretation of between-group differences. The added text reads as follows:

“In addition, the inclusion of a usual-care control group without an attention-matched intervention may have further increased the risk of expectation and attention bias. This factor may be particularly relevant for self-reported outcomes (PDQ-39, BDI) and effort-dependent measures such as the TUG”

**6. Lines 268-274 (Statistical Analysis): The motor subscale (UPDRS-III) was selected as a covariate. Could you justify why the total UPDRS score was not used? Non-motor symptoms are highly relevant to the quality of life and depression outcomes presented here.**

Response: We used UPDRS-III as a covariate because it is a specific, validated measure of motor severity and is the component most directly related to our functional outcomes (TUG and Squat Test). Using the UPDRS total score would have mixed motor and non-motor domains and could have introduced variance not directly relevant to the primary motor/functional outcomes. We have clarified this rationale in the Statistical analysis section and the modified text reads as follows:

“UPDRS-III was selected instead of the UPDRS total score because it provides a specific and validated measure of motor severity, directly linked to functional mobility and strength outcomes. The total score combines motor and non-motor domains, which may reflect broader disease burden rather than motor severity per se.”

**Reviewer #2:**

**Dear Editor,**

**I have reviewed the manuscript entitled “Effects of basic and dual-task training programs on physical function in Parkinson’s disease: the PARKEX study”.
The authors completed the drug therapy with physical training in Parkinson’s disease. The treated groups were compared with a group without physical training. They followed investigation lasted for 12 weeks. The training was applied three times a week. It was a progressive training.
The study is useful, because the effect of training has been shown different from that of drugs. The applied interesting equipment named; “ flywheel device” was used in the course of traditional gymnastic. The description of the training is difficult to understand; I recommend to rewrite it.**

Response: Thank you for your positive evaluation of our study and for highlighting the relevance of combining pharmacological treatment with structured physical training in Parkinson’s disease.

We appreciate your comment regarding the clarity of the training description. The Methods section has been thoroughly revised and restructured to improve clarity, logical flow, and readability. We simplified the explanation of the periodization model, clarified the distinction between BPT and BPT+FE (dual-task condition), and provided a more concise description of intensity progression and task integration. We have also provided a Supplementary Table (see S Table 1) detailing the structure progression of the Dual Task training.

**How many patients with Hoehn-Yahr I, II, III were included in the treated groups?**

Response: As shown in Table 2, all participants included in the study were classified as Hoehn–Yahr stage II. We have clarified this in the text to make the disease severity of the sample more explicit. The new text in the Results section reads as following:

“The sample consisted of 24 participants, all classified as Hoehn–Yahr stage II, reflecting a clinically homogeneous group with mild-to-moderate disease severity.”

**What was the process of dual-task training? Was it applied in the same arrangement for 12 weeks? How many times was it applied?**

Response: The dual-task training was applied three times per week over the 12-week intervention period, following the same overall structure throughout the program. Each session combined flywheel-based resistance exercises with structured cognitive tasks performed simultaneously (dual-task condition). While the session format remained consistent across the 12 weeks, task complexity and motor–cognitive demands were progressively increased according to participants’ performance and adaptation. This progression involved increasing resistance load, modifying coordination demands, and introducing more challenging cognitive tasks to ensure continued stimulus and engagement.

We have clarified these aspects in the revised Methods section to improve transparency and reproducibility of the intervention protocol. We have also provided a Supplementary Table (see S Table 1) detailing the structure progression of the Dual Task training.

**You cited literature of dual-task training, but your results not confirmed them. There has been a publication on a dual-task train for 5 days (J Psychiatry Psychiatric Disord 2023). Can you discuss it comparing with your results?**

**I recommend the manuscript for major revision.**

Response: We acknowledge your concern about the methodological detail of the dual-task intervention. We have substantially expanded the Methods section to provide a clearer and more precise description of the dual-task setting, including task type, structure, progression, frequency, and how cognitive and motor components were combined during training (see Methods section). We have also expanded the Introduction section. The new introduction section reads as follows:

“Given the close interaction between motor and cognitive systems, targeting both domains together offers advantages beyond those achieved with motor training alone. Randomized controlled trials have implemented dual-task interventions combining locomotor activities with concurrent cognitive tasks (e.g., mental arithmetic, verbal fluency) or coordinated motor demands (e.g., obstacle negotiation, manipulative tasks), reporting improvements in mobility, balance, and selected executive domains such as divided attention, inhibitory control, working memory, and set-shifting (e.g., obstacle negotiation, manipulative tasks). [10-16]”

For this, we have added the following new references, including the reference that was suggested in the comment:

10. Dalma Szögedi, Trevor W. Stone, Elek Dinya , Judit Málly. Dual-task performance testing as an indicator of cognitive deterioration in Parkinson's disease: A pilot study. Journal of Psychiatry and Psychiatric Disorders. 7 (2023): 104-117

11. Li Z, Wang T, Liu H, Jiang Y, Wang Z, Zhuang J. Dual-task training on gait, motor symptoms, and balance in patients with Parkinson's disease: a systematic review and meta-analysis. Clin Rehabil. 2020 Nov;34(11):1355-1367.

12. Fernandes, Â., Rocha, N., Santos, R., & Tavares, J. M. R. S. (2015). Effects of dual-task training on balance and executive functions in Parkinson’s disease: A pilot study. *Somatosensory & Motor Research*, *32*(2), 122–127.

13. Salazar RD, Ren X, Ellis TD, Toraif N, Barthelemy OJ, Neargarder S, Cronin-Golomb A. Dual tasking in Parkinson's disease: Cognitive consequences while walking. Neuropsychology. 2017 Sep;31(6):613-623.

14. San Martín Valenzuela C, Moscardó LD, López-Pascual J, Serra-Añó P, Tomás JM. Effects of Dual-Task Group Training on Gait, Cognitive Executive Function, and Quality of Life in People With Parkinson Disease: Results of Randomized Controlled DUALGAIT Trial. Arch Phys Med Rehabil. 2020 Nov;101(11):1849-1856.e1.

15. Wong PL, Cheng SJ, Yang YR, Wang RY. Effects of Dual Task Training on Dual Task Gait Performance and Cognitive Function in Individuals With Parkinson Disease: A Meta-analysis and Meta-regression. Arch Phys Med Rehabil. 2023 Jun;104(6):950-964.

16. Li Z, Wang T, Liu H, Jiang Y, Wang Z, Zhuang J. Dual-task training on gait, motor symptoms, and balance in patients with Parkinson's disease: a systematic review and meta-analysis. Clin Rehabil. 2020 Nov;34(11):1355-1367.

45. Huang YZ, Chang FY, Liu WC, Chuang YF, Chuang LL, Chang YJ. Fatigue and Muscle Strength Involving Walking Speed in Parkinson's Disease: Insights for Developing Rehabilitation Strategy for PD. Neural Plast. 2017;2017:1941980.

**Reviewer #3: The study is interesting and well-written. It is about the study of a potential new intervention method in people in Parkinson. The author adopted a dual-task intervention associated with a resistance training. Despite the topic is interesting and innovative, the methodology is weak and could be implemented and more and precise information on the dual task setting and protocol. Furthermore, important limits are related to the lack of tests related to the cognitive function, also in dual tasking. The TUG alone is not an indicator of the possible effects of the training, a TUG performed in single and dual task could be of great help. Overall, the manuscript requires major revisions. After these revisions are solved, I will proceed with more detailed comments:
-Please, implement the introduction with more information about the dual task concept and the dual task training. There are different reviews on the topic dual task training that could be useful.**

Response: Thank you for your positive comments regarding the originality and relevance of our study. We acknowledge your concern about the methodological detail of the dual-task intervention. We have substantially expanded the Methods section to provide a clearer and more precise description of the dual-task setting, including task type, structure, progression, frequency, and how cognitive and motor components were combined during training (see Methods section). We have also expanded the Introduction section. The new introduction section reads as follows:

“Given the close interaction between motor and cognitive systems, targeting both domains together offers advantages beyond those achieved with motor training alone. Randomized controlled trials have implemented dual-task interventions combining locomotor activities with concurrent cognitive tasks (e.g., mental arithmetic, verbal fluency) or coordinated motor demands (e.g., obstacle negotiation, manipulative tasks), reporting improvements in mobility, balance, and selected executive domains such as divided attention, inhibitory control, working memory, and set-shifting (e.g., obstacle negotiation, manipulative tasks). [10-16]”

For this, we have added the following new references:

10. Dalma Szögedi, Trevor W. Stone, Elek Dinya , Judit Málly. Dual-task performance testing as an indicator of cognitive deterioration in Parkinson's disease: A pilot study. Journal of Psychiatry and Psychiatric Disorders. 7 (2023): 104-117

11. Li Z, Wang T, Liu H, Jiang Y, Wang Z, Zhuang J. Dual-task training on gait, motor symptoms, and balance in patients with Parkinson's disease: a systematic review and meta-analysis. Clin Rehabil. 2020 Nov;34(11):1355-1367.

12. Fernandes, Â., Rocha, N., Santos, R., & Tavares, J. M. R. S. (2015). Effects of dual-task training on balance and executive functions in Parkinson’s disease: A pilot study. *Somatosensory & Motor Research*, *32*(2), 122–127.

13. Salazar RD, Ren X, Ellis TD, Toraif N, Barthelemy OJ, Neargarder S, Cronin-Golomb A. Dual tasking in Parkinson's disease: Cognitive consequences while walking. Neuropsychology. 2017 Sep;31(6):613-623.

14. San Martín Valenzuela C, Moscardó LD, López-Pascual J, Serra-Añó P, Tomás JM. Effects of Dual-Task Group Training on Gait, Cognitive Executive Function, and Quality of Life in People With Parkinson Disease: Results of Randomized Controlled DUALGAIT Trial. Arch Phys Med Rehabil. 2020 Nov;101(11):1849-1856.e1.

15. Wong PL, Cheng SJ, Yang YR, Wang RY. Effects of Dual Task Training on Dual Task Gait Performance and Cognitive Function in Individuals With Parkinson Disease: A Meta-analysis and Meta-regression. Arch Phys Med Rehabil. 2023 Jun;104(6):950-964.

16. Li Z, Wang T, Liu H, Jiang Y, Wang Z, Zhuang J. Dual-task training on gait, motor symptoms, and balance in patients with Parkinson's disease: a systematic review and meta-analysis. Clin Rehabil. 2020 Nov;34(11):1355-1367.

45. Huang YZ, Chang FY, Liu WC, Chuang YF, Chuang LL, Chang YJ. Fatigue and Muscle Strength Involving Walking Speed in Parkinson's Disease: Insights for Developing Rehabilitation Strategy for PD. Neural Plast. 2017;2017:1941980.

**-Please, implement the details of the dual task within the methods. The videos are not enough, a better explanation of the dual task training is required.**

Response: The Methods section has been thoroughly revised and restructured to improve clarity, logical flow, readability, and reproducibility. A detailed description of the dual-task process has been incorporated, specifying when and how the dual-task activities were implemented within each session (warm-up, transitions/active rest, and cool-down), their total duration (12–15 minutes per session), the week of introduction (week 5), and their mesocycle-based progression. Importantly, flywheel-based eccentric resistance training and dual-task activities were performed within the same session but not simultaneously. We have also provided a Supplementary Table (see S Table 1) detailing the structure progression of the Dual Task training.

**-Please, highlight in the limits of the study that no test on the cognitive function have been performed. It is important to evaluate not only the aspects related to questionnaire, but according to my opinion, more information about the cognitive health could be helpful in better understand how the intervention work in this population*.***

Response: Global cognitive function was assessed using the Montreal Cognitive Assessment (MoCA), a cognitive screening tool, at baseline and post-intervention. No significant changes were observed between groups. As cognitive outcomes were not a primary focus of the study and the trial was not powered to detect changes in global cognition, these results were not emphasized. Moreover, MoCA, as a screening instrument, may not be sufficiently sensitive to detect subtle executive-function adaptations associated with dual-task training. We have now clarified this point in the manuscript modifying the section:

“Effects of exercise on overall satisfaction, quality of life and cognitive function”

And adding the following text:

“No significant between-group differences were observed in global cognitive function (MoCA).”
